# Effects of root and leaf litter identity and diversity on oribatid mite abundance, species richness and community composition

**DOI:** 10.1371/journal.pone.0219166

**Published:** 2019-07-10

**Authors:** Christian Bluhm, Olaf Butenschoen, Mark Maraun, Stefan Scheu

**Affiliations:** 1 University of Göttingen, J.F. Blumenbach Institute of Zoology and Anthropology, Göttingen, Germany; 2 University of Göttingen, Centre of Biodiversity and Sustainable Land Use, Göttingen, Germany; Helmholtz Zentrum Munchen Deutsches Forschungszentrum fur Umwelt und Gesundheit, GERMANY

## Abstract

Habitat heterogeneity is an important driver of aboveground species diversity but few studies have investigated effects on soil communities. Trees shape their surrounding by both leaf litter and roots generating small scale heterogeneity and potentially governing community patterns of soil organisms. To assess the role of vegetation for the soil fauna, we studied whether tree species (*Fagus sylvatica* L., *Acer pseudoplatanus* L., *Fraxinus excelsior* L., *Tilia cordata* Mill.), markedly differing in leaf litter quality and root associated mycorrhizal symbionts, affect oribatid mite communities by shaping below- and aboveground resources and habitat complexity and availability. Oribatid mite abundance, species richness, community structure and the proportion of litter living and parthenogenetic individuals were analyzed and related to microbial biomass and the amount of remaining litter mass. Although leaf litter species with higher nutritional values decomposed considerably faster, microbial biomass only slightly differed between leaf litter species. Neither root species nor leaf litter species affected abundance, species richness or community structure of oribatid mites. However, root species had an effect on the proportion of parthenogenetic individuals with increased proportions in the presence of beech roots. Overall, the results suggest that identity and diversity of vegetation via leaf litter or roots are of minor importance for structuring oribatid mite communities of a temperate forest ecosystem.

## Introduction

Habitat heterogeneity is an important driver of species diversity [1–4]. The increase in species numbers in complex environments is based on the assumption that the more heterogeneous a habitat the more potential niches are present. Although the majority of species live permanently or periodically in soil, e.g. as larval stages [5,6], most studies dealing with the role of habitat heterogeneity for species diversity investigated aboveground communities and focused on large species such as vertebrates [1]. Despite the seemingly homogenous habitat, species richness in soil is extraordinary high, especially at small spatial scales of meters or centimeters. One square meter of forest soil can be inhabited by hundreds of thousands of individuals and species of metazoans next to a myriad of microorganisms [5,7–9]. This high diversity may be due to small-scale heterogeneity governing these communities locally [10–12].

Similar to aboveground habitats, belowground habitats are shaped mainly by plants. Since quality and quantity of litter and roots varies among plant species, a greater plant diversity is generally assumed to promote food and habitat complexity, thereby fostering microbial and animal diversity [13–15]. Leaf litter markedly differs among plant species in physical and chemical traits including structural compounds, secondary metabolites and nutrients shaping the upper soil layers [16,17]. Leaf litter quality thus may have a considerable impact on soil organisms living in and feeding on litter. For example, microbial communities vary markedly between litter species of different quality, but also within the same litter species at different stages of decomposition [18–21]. In the soil, plant roots structure the environment by forming pores of different size and by providing root-exudates which are increasingly recognized as major factor structuring belowground communities [22–25]. In addition, most plants are associated with mycorrhizal fungi which reach substantial biomass in soil and thereby may represent an important food resource for soil animals [26–29]. Although the exact mechanisms are still unclear, plant roots heavily impact soil animal communities with their role potentially even outweighing that of leaf litter [24,30,31].

Oribatid mites are one of the most widespread, abundant and diverse soil animals that predominantly inhabit the litter and the upper soil layer where they mainly feed on dead organic matter and fungi [32–35]. Their abundance and diversity generally increases from agricultural sites to grasslands and reach a maximum in forest soils where organic material accumulates [36,37]. Simultaneously to the increase in abundance, the proportion of oribatid mite species reproducing parthenogenetically has been shown to increase with the thickness of the litter layer [38]. In forests, communities of oribatid mites often differ markedly between patches even at short distances [39], presumably due to small-scale habitat and resource heterogeneity mediated by plant species identity and community composition [5,40–42]. However, only few attempts have been made to disentangle the relative contribution of leaf litter and roots of different plant species on soil animal communities; and in existing studies on oribatid mite communities effects of leaf litter range from being strong [40,41,43,44], weak [45–47] or even absent [48–50]. Even rarer are studies on the effect of roots on oribatid mite communities and the results of the few existing studies also are ambiguous [51,52].

In the present study we investigated how leaf litter and roots of different tree species of deciduous forests (*Fagus sylvatica* L., *Acer pseudoplatanus* L., *Fraxinus excelsior* L., *Tilia cordata* Mill.) influence local microbial biomass, and abundance, species richness, proportion of parthenogenetic individuals and the community structure of oribatid mites in a field experiment. We further tested whether mixtures of leaf litter and roots of these tree species alter community structure as compared to single litter and root species treatments. The four tree species were chosen because they represent common species in Central European forests and differ in leaf litter as well as in root characteristics. Beech produces low, whereas maple, lime and ash produce high quality litter reflected e.g., by their C/N ratio [53–55]. The root systems of beech and lime are associated with ectomycorrhizal fungi (EMF) while those of ash and maple are associated with arbuscular mycorrhizal fungi (AMF).

We hypothesized that (1) low quality litter harbors higher densities of oribatid mites than easily degradable leaf litter since leaf litter not only serves as a resource but also as a habitat and this vanishes in fast decomposing litter. As the relative abundance of parthenogenetic oribatid mites has been shown to increase with litter thickness, we hypothesized that (2) it will be higher in recalcitrant litter than in easily decomposable high quality litter. Further, we hypothesized that (3) oribatid mite species richness is highest in litter and root mixtures as it increases resource and structural heterogeneity facilitating the coexistence of species. In addition, we hypothesized that due to structural and chemical differences (4) leaf litter and root species identity affect oribatid mite community composition.

## Materials and methods

### Study site and design

The study formed part of the “SPLIDRHEX”-project (**Sp**ecies **l**itter **i**dentity and **d**iversity effects on the **rh**izosphere of trees **ex**periment) which was established in an old-growth deciduous forest near Göttingen (51°26‘27‘‘N, 10°01‘03‘‘E, 340 m a.s.l., Lower Saxony, Germany). The forest grows on oligotroph brown earth of a pH (CaCl_2_) of 5.01 ± 0.07 with mull humus and is dominated by sessile oak (*Quercus petraea* L.) interspersed with European beech (*F*. *sylvatica*). The understory is species-rich and dominated by jewelweed (*Impatiens* spp.), stinging-nettle (*Urtica dioica* L.) and fern (*Athyrium filix-femina* (L.) Roth). Soil carbon and nitrogen concentrations are 2.22 ± 0.05% and 0.14 ± 0.003%, respectively. The bedrock consists of red sandstone. Mean annual temperatures and mean annual precipitation are 8.7°C and 644 mm, respectively.

In November 2010, four adjacent blocks with each 25 plots being at least 100 cm apart were set up at the forest site. In each plot either single tree species including beech (*F*. *sylvatica*), maple (*A*. *pseudoplatanus*), ash *(F*. *excelsior*), lime (*T*. *cordata*) or a mixture of all four tree species were planted (hereafter referred to as root species treatment). Each plot received either one of the single leaf litter species from the four tree species mentioned above or a mixture of all four leaf litter species (hereafter referred to as leaf litter species treatments) resulting in 25 different combinations of root and leaf litter species plots.

Each plot (180 x 210 cm) comprised 30 trees equidistantly arranged in a square 5 x 6 design. The trees were planted in spring 2011 as two year old saplings with an approximate height of 20 cm. Prior to planting, the original leaf litter layer was removed and replaced by 800 g air-dried leaf litter of the respective tree species in the single leaf litter treatments or by equal parts of each leaf litter species in the litter mixture treatments. The litter used for this experiment was collected by litter traps in monospecific forests in the vicinity of Göttingen. To prevent leaf litter loss by wind and mixture with non-target litter from the surrounding, the plots were covered by nets of a mesh size of 1 cm. The litter was restocked every year by adding 800 g of the respective litter species / mixture in autumn mimicking natural annual litter fall. The amount of litter added resembles the amount of litter entering the soil with the annual litter fall in deciduous forests. As indicated by nitrogen concentrations litter quality of lime (11.2 ± 0.20 mg/g dry mass, C-to-N ratio 40.5), maple (11.1 ± 0.20 mg/g dry mass, C-to-N ratio 36.6) and ash (10.3 ± 0.20 mg/g dry mass, C-to-N ratio 40.2) was similar and higher than that of beech (7.8 ± 0.10 mg/g dry mass, C-to-N ratio 57.5). More details on litter chemistry are given in Yang et al. [56].

### Sampling procedure

In November 2015, one soil sample were taken from each plot using a soil corer (Ø 5 cm) and separated into litter and soil layer (depth of 5 cm). Animals were extracted by heat using a high gradient extractor and were stored in 70% ethanol until determination [57]. Oribatid mites were determined to species level except for Brachychthoniidae, *Suctobelbella* and *Phthiracarus* which were determined to family and genus level, respectively, using the key of Weigmann [58]. Juvenile oribatid mites were counted. Information on the reproductive mode was taken from the literature [59–63]. Litter and soil layers were fused for the statistical analysis but information on the horizontal distribution of oribatid mites was maintained by calculating the proportion of litter living individuals from the total abundance of both layers. For measurements of remaining leaf litter mass, additional samples were taken with a soil corer of a diameter of 21 cm. The litter was dried at 50°C, cleaned and weighed. Results on remaining leaf litter mass were extrapolated to plot area (3.78 m^2^) to allow comparison with the amount added annually.

For analysis of microbial biomass, three samples were taken from each plot, divided into litter and soil layer and then pooled. Samples were homogenized by sieving the soil through 2 mm mesh and by cutting the litter with scissors into small fragments. Microbial biomass was measured by substrate-induced respiration (SIR) after addition of 8 and 80 mg glucose g^−1^ dry weight to soil and litter, respectively [64,65]. Microbial respiration was determined by measuring oxygen consumption using an automated system based on electrolytic oxygen microcompensation [66]. The mean of the three lowest readings per hour within 2–10 h after addition of glucose was taken as the maximum initial respiratory response (MIRR). Microbial biomass (C_mic_) was calculated as 38×MIRR [67].

No permission was needed for taking the soil samples as they did not include any endangered or protected species.

### Statistical analysis

Remaining litter mass, microbial biomass and oribatid mite abundance, species richness, proportion of litter living individuals and proportion of parthenogenetic individuals were analyzed by two-way analysis of variance (ANOVA) with the fixed factors leaf litter species (ash, beech, lime, maple, mixture) and root species (ash, beech, lime, maple, mixture), and block (1, 2, 3, 4) as random factor using R v 3.4.3 (R Development Core Team 2017). Since microbial biomass was more than 10-fold higher in the litter than in the soil layer both layers were analyzed separately. Differences between means were inspected by Tukey’s honestly significant difference (HSD) post-hoc test. Linear regressions of oribatid mite abundance, species richness, proportion of litter living and parthenogenetic individuals with remaining litter mass and microbial biomass were conducted using R. To improve normality and homoscedasticity, data on oribatid mite abundance, microbial biomass and remaining litter mass were log-transformed.

To inspect for effects of leaf litter and root identity and diversity on oribatid mite community structure the dataset was analyzed by multivariate analysis of variance (MANOVA) using R. Prior to the MANOVA the dataset containing 46 species was reduced by non-metric multidimensional scaling (NMDS) using the ‘vegan’ package v 2.4–6 implemented in R [68]. Stress value was below the recommended 0.05 threshold at 13 dimensions (0.047) and thus was taken for MANOVA.

Redundancy analysis (RDA) was carried out in Canoco 5.02 (Microcomputer Power, Ithaca, New York; [69]) with all oribatid mite species occurring in more than 3 samples. Litter and root species were implemented as supplementary and microbial biomass (C_mic_) and remaining litter mass as environmental variables.

## Results

**Fig 1:**
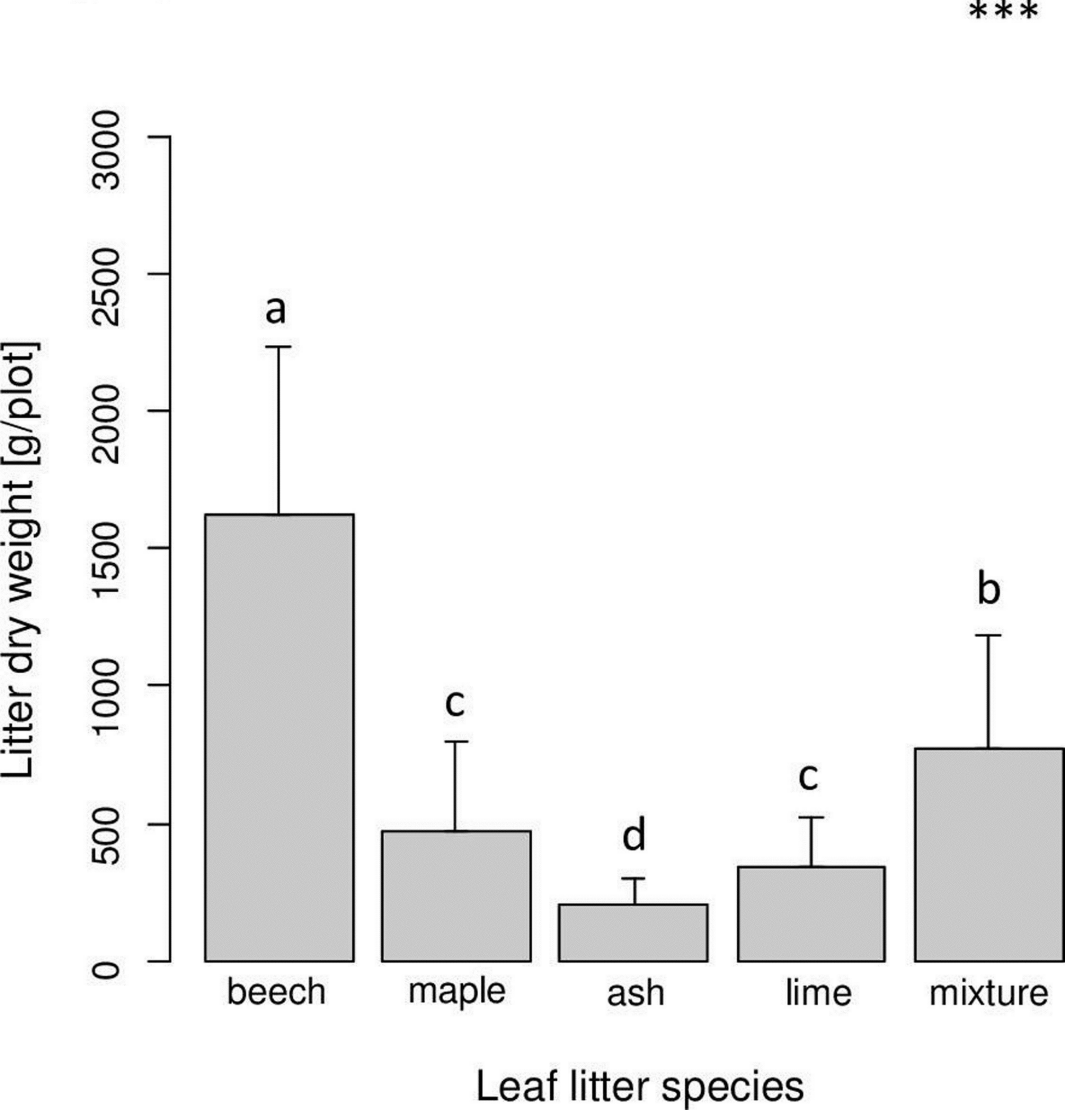
Variation in remaining leaf litter mass with leaf litter species of beech, maple, ash and lime; mixture refers to the mixture of the four leaf litter species. Bars sharing the same letter do not differ significantly (Tukey’s HSD test, p < 0.05).

Remaining litter mass varied significantly with leaf litter species but was independent of root species (Tables 1 and 2). Beech leaf litter by far decomposed slowest followed by maple, lime and ash (Fig 1). Remaining litter mass of the leaf litter mixture was intermediate and similar to the mean of the four single leaf litter species.

**Table 1:** Effects of leaf litter and root species on remaining litter mass, microbial biomass in litter and soil, oribatid mite abundance, species richness and the proportion of parthenogenetic and litter living individuals.

	Leaf litter species		Root species		Root species x Leaf litter species	
	p	F_4,72_	p	F_4,72_	p	F_16,72_
Remaining litter mass (g/plot)	**<0.001**	**41.02**	0.681	0.58	0.994	0.32
Microbial biomass litter (mg C_mic_/g litter dw)	0.067	2.31	0.294	1.26	0.520	0.95
Microbial biomass soil (mg C_mic_/g soil dw)	**0.001**	**5.07**	0.096	2.06	0.478	0.99
Abundance (ind./m^2^)	0.912	0.24	0.299	1.25	0.565	0.91
Species richness (species/sample)	0.867	0.31	0.324	1.19	0.322	1.16
Litter living ind. (%)	0.053	2.46	0.549	0.77	0.818	0.66
Parthenogentic ind. (%)	0.370	1.08	**<0.001**	**5.19**	0.110	1.54

Significant values are marked in bold.

**Table 2:** Means (± SD) of remaining litter mass (g/plot), microbial biomass in the litter and soil layer (mg C_mic_/g soil dw), oribatid mite abundance (ind./m^2^), species richness (species/sample) and the proportion of litter living and parthenogenetic individuals (%) in soil samples of different leaf litter and root species.

		Leaf litter species																	
		maple			beech			ash			lime			mixture			average		
		mean		sd	mean		sd	mean		sd	mean		sd	mean		sd	mean		sd
Remaining litter mass																			
Root species	beech	444.3	±	339.1	1609.2	±	407.0	212.1	±	102.3	293.7	±	144.5	789.7	±	397.4	714.5	±	616.8
	maple	445.4	±	554.3	1781.8	±	327.0	242.4	±	133.6	419.2	±	216.0	731.3	±	360.9	668.4	±	606.8
	ash	510.1	±	392.5	1965.5	±	1208.2	178.3	±	87.9	410.5	±	234.0	824.9	±	594.8	777.8	±	858.6
	lime	455.8	±	217.0	1298.1	±	416.4	197.6	±	60.5	237.2	±	181.5	788.1	±	207.5	601.9	±	471.0
	mixture	488.2	±	201.9	1495.6	±	355.6	197.9	±	112.8	348.0	±	108.6	715.5	±	631.9	642.6	±	561.1
	average	468.8	±	324.9	1621.4	±	615.1	205.7	±	93.6	341.7	±	177.3	769.9	±	415.9			
Microbial biomass (litter layer)																			
Root species	beech	10.07	±	3.19	9.43	±	4.43	14.09	±	6.79	11.45	±	3.57	13.86	±	4.51	11.75	±	4.44
	maple	9.07	±	3.99	12.97	±	7.05	14.15	±	4.65	19.52	±	7.82	10.75	±	4.92	12.96	±	6.05
	ash	12.60	±	4.11	11.99	±	4.02	15.32	±	4.34	11.51	±	1.46	9.39	±	2.32	12.32	±	3.61
	lime	11.43	±	3.87	11.30	±	1.95	14.14	±	5.91	11.35	±	1.00	13.75	±	8.04	12.38	±	4.33
	mixture	14.92	±	7.74	14.70	±	4.01	17.31	±	5.70	13.13	±	4.76	13.15	±	6.35	14.72	±	5.37
	average	11.70	±	4.85	12.08	±	4.38	15.05	±	4.98	13.07	±	4.68	12.19	±	5.04			
Microbial biomass (soil layer)																			
Root species	beech	0.85	±	0.26	0.74	±	0.18	0.86	±	0.18	0.69	±	0.29	0.74	±	0.41	0.78	±	0.25
	maple	0.76	±	0.19	0.70	±	0.28	0.90	±	0.09	0.78	±	0.13	0.53	±	0.18	0.73	±	0.21
	ash	0.93	±	0.13	0.67	±	0.19	1.18	±	0.44	0.87	±	0.28	0.70	±	0.11	0.87	±	0.30
	lime	0.69	±	0.19	0.67	±	0.16	0.79	±	0.12	0.55	±	0.14	0.74	±	0.14	0.69	±	0.16
	mixture	0.88	±	0.17	0.61	±	0.06	0.74	±	0.10	0.58	±	0.09	0.68	±	0.19	0.70	±	0.16
	average	0.83	±	0.19	0.68	±	0.17	0.89	±	0.25	0.68	±	0.22	0.68	±	0.22			
**Oribatida**																			
Abundance																			
Root species	beech	39,089	±	39,691	55,921	±	41,301	31,067	±	29,398	45,582	±	19,595	39,216	±	18,311	42,829	±	29,874
	maple	36,669	±	19,659	38,027	±	16,076	71,811	±	59,076	44,818	±	11,350	24,064	±	18,756	43,344	±	32,135
	ash	35,906	±	20,660	33,741	±	9,513	29,157	±	12,745	43,927	±	23,143	38,707	±	16,154	36,287	±	16,085
	lime	46,473	±	27,472	20,754	±	11,391	40,489	±	37,995	25,847	±	19,753	39,343	±	20,322	34,581	±	24,344
	mixture	40,871	±	26,925	23,810	±	15,298	30,176	±	37,945	27,884	±	24,142	25,592	±	9,038	29,667	±	22,837
	average	39,802	±	25,015	35,345	±	25,376	40,540	±	37,988	37,612	±	20,023	33,384	±	16,717			
Species richness																			
Root species	beech	7.5	±	4.4	9.0	±	2.2	6.0	±	2.6	8.0	±	1.4	7.0	±	0.0	7.6	±	2.5
	maple	6.5	±	2.4	9.3	±	1.5	9.0	±	2.9	9.3	±	2.1	6.0	±	2.2	7.9	±	2.5
	ash	7.3	±	2.6	9.5	±	1.9	7.5	±	2.5	8.5	±	1.9	11.0	±	4.1	8.8	±	2.8
	lime	8.5	±	3.1	6.0	±	1.4	8.0	±	6.3	6.8	±	3.3	8.0	±	1.8	7.5	±	3.4
	mixture	8.8	±	3.9	6.0	±	2.2	5.5	±	3.9	6.3	±	2.6	8.8	±	3.3	7.1	±	3.2
	average	7.7	±	3.1	8.0	±	2.4	7.2	±	3.7	7.8	±	2.4	8.2	±	2.9			
Litter living individuals																			
Root species	beech	48.0	±	16.4	49.0	±	16.9	41.6	±	8.4	60.6	±	15.5	73.4	±	17.3	54.2	±	17.8
	maple	63.7	±	24.0	62.5	±	25.6	49.5	±	30.9	73.7	±	18.2	63.4	±	25.7	62.6	±	23.6
	ash	60.5	±	30.0	66.5	±	8.9	50.2	±	25.5	80.2	±	11.2	59.6	±	13.8	63.4	±	20.2
	lime	58.0	±	18.0	62.7	±	20.1	58.2	±	12.5	59.8	±	5.0	51.9	±	6.5	58.1	±	12.8
	mixture	53.9	±	10.9	69.7	±	19.7	47.7	±	25.9	58.0	±	14.7	63.4	±	9.2	58.5	±	17.1
	average	56.8	±	19.4	61.4	±	18.1	49.5	±	20.6	66.5	±	15.2	62.3	±	15.9			
Parthenogenetic individuals																			
Root species	beech	27.8	±	13.4	58.3	±	27.1	69.0	±	18.1	45.7	±	32.6	43.1	±	19.5	49.2	±	25.3
	maple	38.9	±	18.1	63.1	±	10.4	42.3	±	42.9	24.5	±	21.3	45.3	±	27.5	41.7	±	26.9
	ash	21.4	±	10.8	37.6	±	27.3	33.1	±	15.9	32.5	±	32.2	42.7	±	21.5	33.5	±	21.5
	lime	37.3	±	35.8	16.3	±	18.5	14.4	±	16.8	14.6	±	10.1	39.0	±	22.1	24.3	±	23.0
	mixture	43.4	±	23.5	21.1	±	6.1	8.0	±	9.3	24.7	±	20.4	33.7	±	14.6	26.2	±	18.9
	average	33.7	±	21.3	39.0	±	26.6	40.8	±	19.5	28.4	±	24.4	40.8	±	19.5			

**Fig 2:**
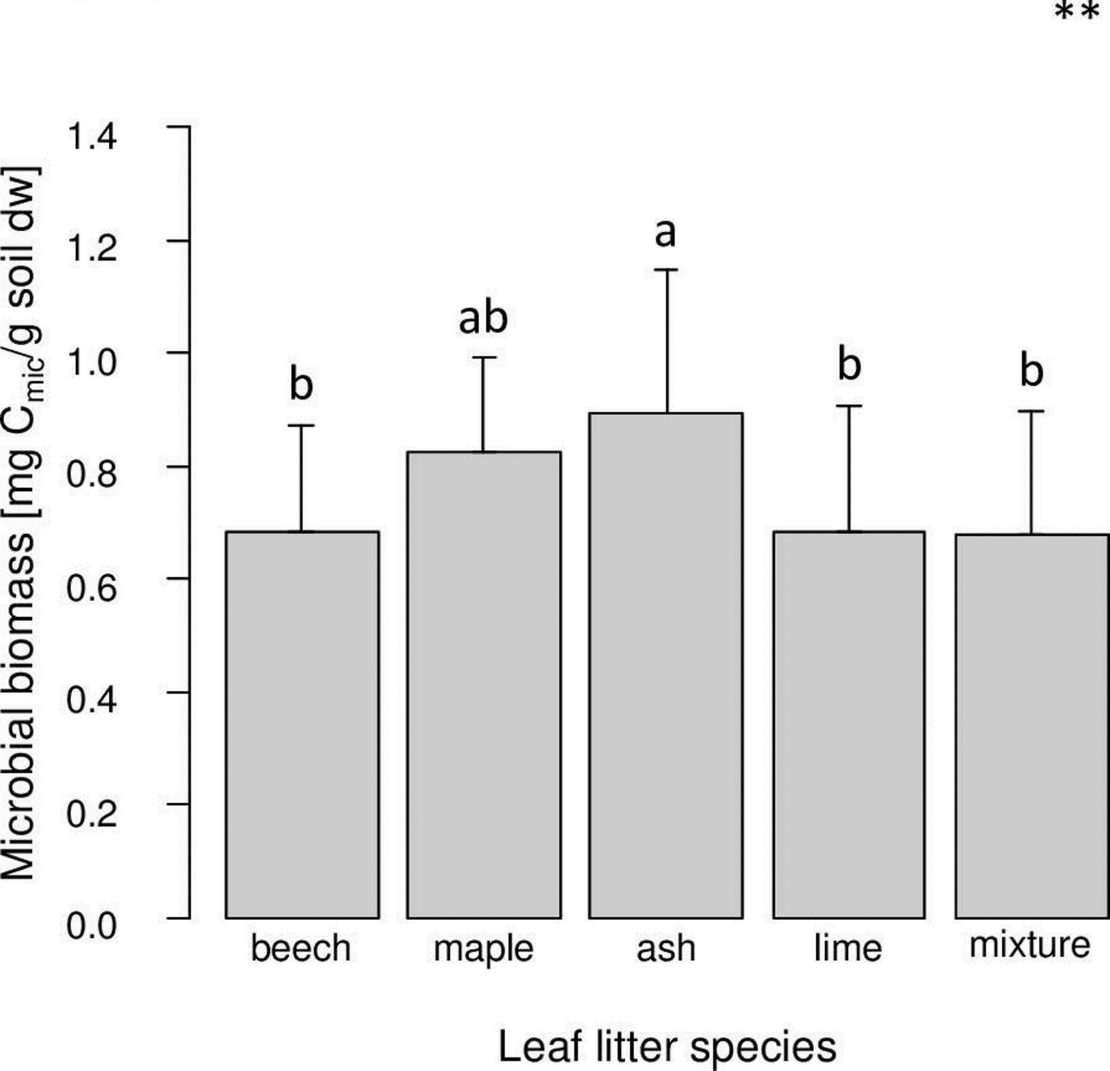
Variations in microbial biomass in soil with leaf litter species of beech, maple, ash and lime; mixture refers to the mixture of the four leaf litter species. Bars sharing the same letter do not differ significantly (Tukey’s HSD test, p < 0.05).

In the soil layer, microbial biomass was significantly higher in the presence of ash leaf litter as compared to those of beech, lime and the mixture (Fig 2 and Tables 1 and 2). Similarly, microbial biomass in the litter layer tended to be higher in ash as compared to the other leaf litter species and the mixture.

In total, 7330 oribatid mite individuals were determined of which 3875 were adult. The adult individuals included 45 species, eight of them singletons. The community was dominated by species of the family Oppiidae and Suctobelbidae that accounted for more than 60% of the individuals; other abundant families included Phthiracaridae (8.9%), Achipteriidae (8.8%), Brachychthoniidae (7.3%) and Chamobatidae (6.9%), altogether representing more than 90% of total adult oribatid mites (S1 Appendix).

Oribatid mite abundance neither differed between leaf litter species nor between root species treatments (Tables 1 and 2). Abundance also neither correlated with remaining litter mass (F_1,98_ = 0.42, p = 0.518; R^2^ = 0.004) nor with microbial biomass in the soil layer (F_1,94_ = 2.33, p = 0.130; R^2^ = 0.024) but it tended to decrease with microbial biomass in the litter layer (F_1,89_ = 3.72, p = 0.057; R^2^ = 0.040).

Oribatid mite species richness per sample neither differed between leaf litter species nor between root species treatments (Tables 1 and 2). However, species richness was positively correlated with remaining litter mass (F_1,98_ = 4.58, p = 0.035; R^2^ = 0.044). Further, oribatid mite species richness neither correlated with microbial biomass in the soil (F_1,94_ = 0.50, p = 0.482; R^2^ = 0.005) nor with microbial biomass in the litter layer (F_1,89_ = 0.96, p = 0.329; R^2^ = 0.011).

The proportion of litter living oribatid mites tended to be lower in ash as compared to the other three single leaf litter species and the litter mixture, but did not differ significantly between root species (Tables 1 and 2). The proportion of litter living oribatid mites neither correlated with microbial biomass in soil (F_1,94_ = 0.02, p = 0.9; R^2^ = 0.0002) nor with microbial biomass in the litter layer (F_1,89_ = 1.14, p = 0.288; R^2^ = 0.013), but correlated positively with remaining litter mass (F_1,98_ = 5.75, p = 0.018; R^2^ = 0.055).

**Fig 3:**
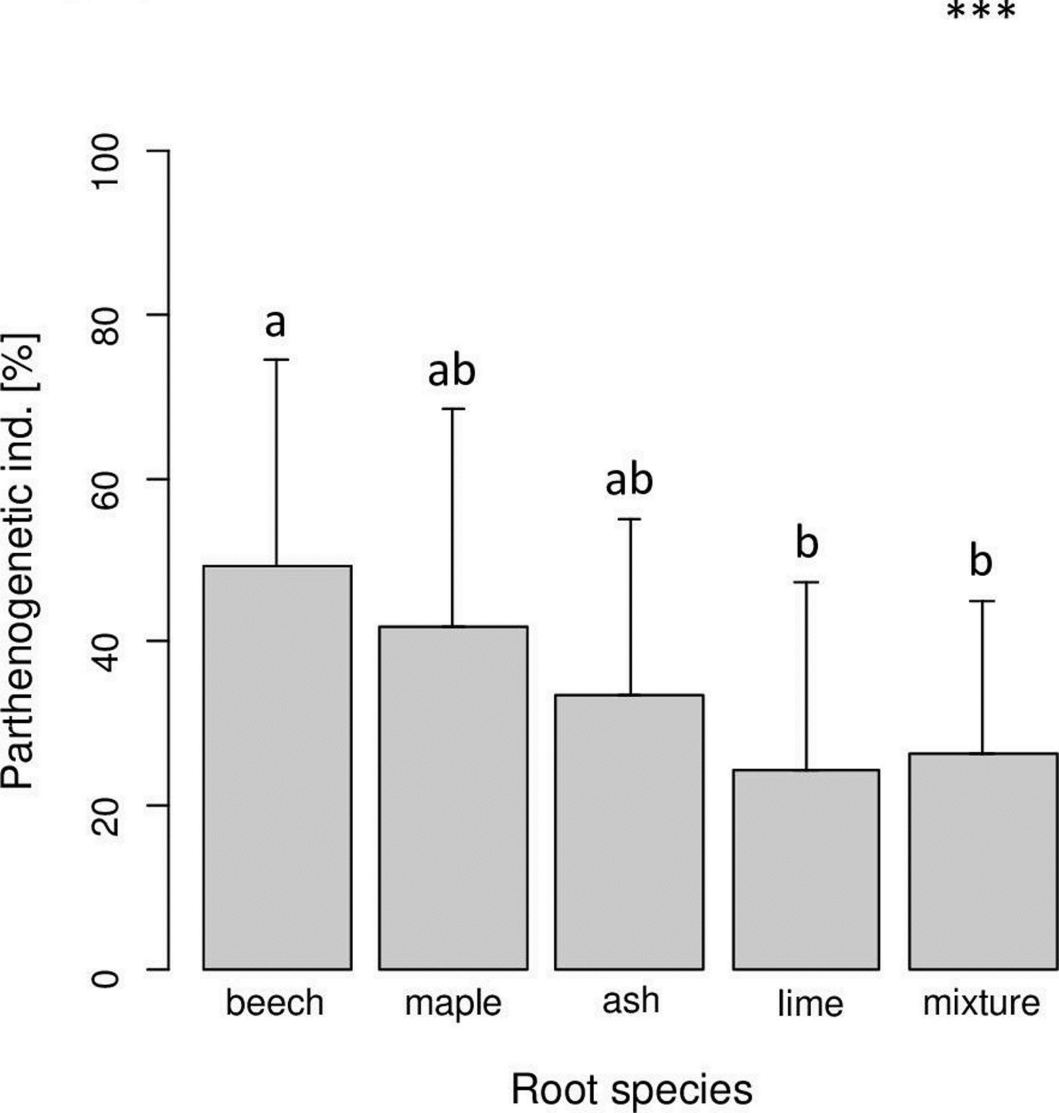
Variations in the proportion of parthenogenetic oribatid mite individuals with root species of beech, maple, ash and lime; mixture refers to the mixture of the four root (tree) species. Bars sharing the same letter do not differ significantly (Tukey’s HSD test, p < 0.05).

The percentage of parthenogenetic oribatid mite individuals was not significantly affected by leaf litter species (Tables 1 and 2). However, it significantly differed between root species with values increasing from lime to mixture to ash to maple to beech (Fig 3). The percentage of parthenogenetic oribatid mite individuals was negatively correlated with microbial biomass in the litter layer (F_1,89_ = 7.03, p < 0.001; R^2^ = 0.073) and in trend positively with remaining litter mass (F_1,98_ = 3.59, p = 0.06; R^2^ = 0.035).

**Fig 4:**
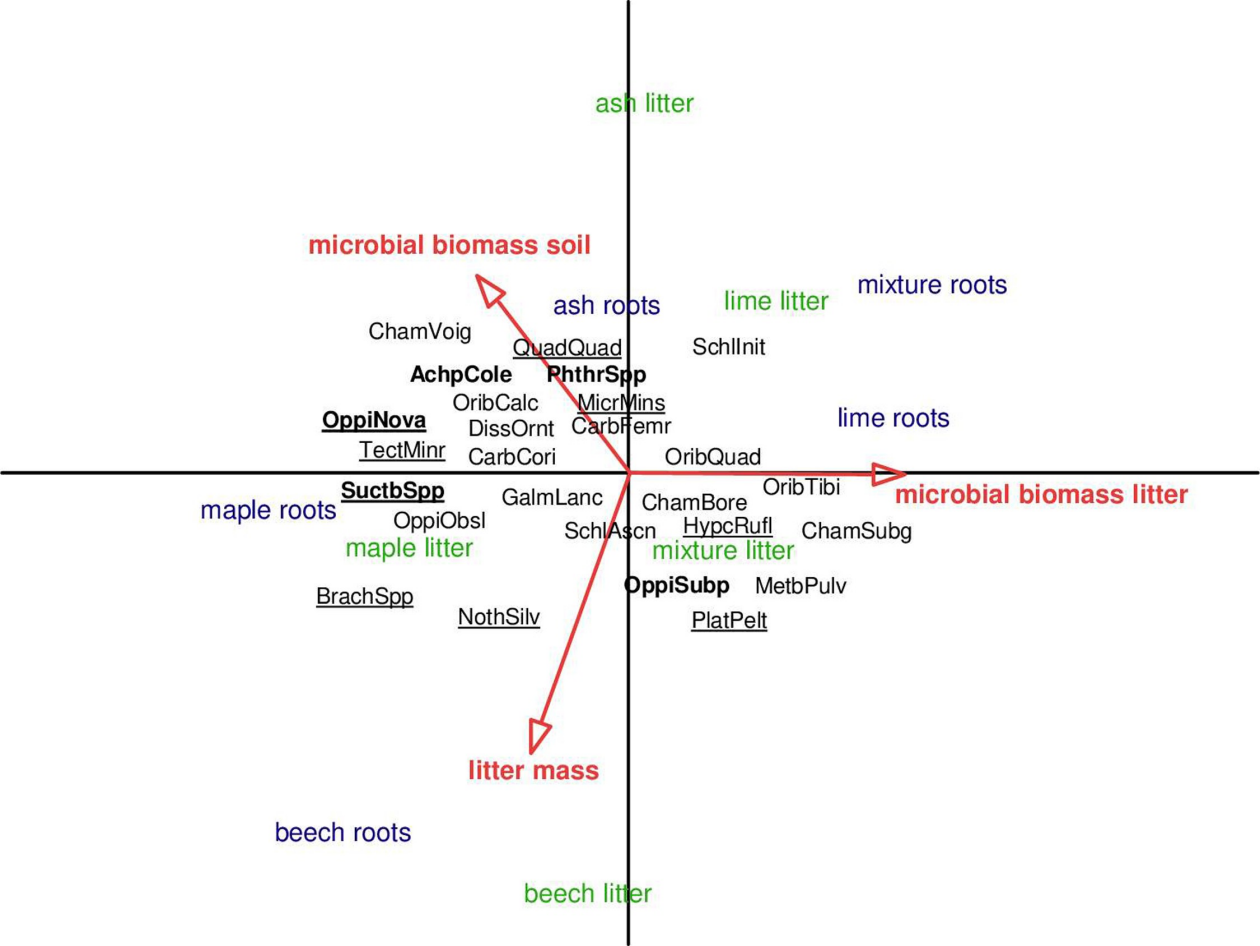
Redundancy analysis (RDA) of oribatid mite species of different leaf litter and root species treatments. Microbial biomass in litter and soil as well as remaining litter mass were included as environmental variables. Litter (green) and root treatments (blue) were included as silent variables not affecting the ordination. Only oribatid mite species occurring in more than three samples were included. Length of gradient 2.4; eigenvalues of 0.053 and 0.014 for the first and second axis, respectively. The five most abundant taxa are marked in bold. Parthenogenetic species are underlined. For full names of species see S1 Appendix.

The structure of oribatid mite communities neither differed significantly between leaf litter (MANOVA; Wilks’ lambda 0.56, F_4,91_ = 1.05, p = 0.39) nor between root species treatments (MANOVA; Wilks’ lambda 0.57, F_4,91_ = 1.03, p = 0.43, Fig 4). Microbial biomass in litter and soil and remaining litter mass significantly affected oribatid mite community composition (RDA; eigenvalues of axis 1 = 0.053 and axis 2 = 0.014; pseudo-F = 2.2, p = 0.004), e.g. Brachychthoniidae, *Nothrus silvestris* Nicolet, 1855 and *Platynothrus peltifer* (C.L. Koch, 1839) were associated with thick litter layers.

## Discussion

We investigated the response of oribatid mites to leaf litter and roots of four tree species (ash, beech, lime and maple) strongly differing in leaf litter quality and root-associated mycorrhizal symbionts. We found little evidence that trees, neither via their leaf litter nor via their roots, impact oribatid mite abundance, species richness or community structure five years after implementing the manipulation.

Leaf litter quality of the different tree species was reflected in the remaining litter mass being highest in beech and lowest in ash litter; and this effect was independent of root species. Generally, decomposition of the four leaf litter species followed initial nitrogen concentrations of the litter species (see Methods; [53–56]). Remaining litter mass of beech leaf litter and the leaf litter mixture (including beech leaf litter) was higher than the amount of leaf litter added per year, suggesting that beech leaf litter accumulated with time. By contrast, leaf litter of the other tree species (with higher nitrogen concentrations) almost completely disappeared within one year suggesting high nutritional value for detritivores. In part this was corroborated by microbial biomass which was significantly higher in the soil and by trend also in the litter layer when ash litter was present. However, microbial biomass in the litter as well as in the soil layer was similar among the three other leaf litter species, although nitrogen concentrations of maple and lime litter resembled that of ash litter and exceeded that in beech litter (see Methods). Mixing of litter did not affect microbial biomass as has been reported in previous studies [19,20]. Likewise, despite marked differences in leaf litter decomposition rates, abundances of oribatid mites were similar in each of the single leaf litter species treatments and in the litter mixture, suggesting that detritivore animals did not benefit from high leaf litter quality. In a previous study, Anderson [48] also found similar densities of oribatid mites in fast decomposing litter of sweet chestnut and slow decomposing litter of beech, and argued that the nutritional value of litter is of minor importance. By contrast, Wardle et al. [70] and Eissfeller et al. [44] found the density of oribatid mites in different leaf litter species to vary, but also differences were not related to leaf litter quality as indicated by nitrogen concentrations. However, potential positive effects of higher litter quality might have been counteracted by habitat loss, i.e. fast litter decomposition.

In contrast to our third hypothesis, neither more diverse leaf litter (four leaf litter species) nor more diverse roots (four tree species) significantly increased oribatid mite species richness as compared to the respective single species treatments. This is conform to earlier studies and indicates that higher leaf litter diversity is not increasing habitat complexity of oribatid mites ([42,47,48,68]; but see [38,40]). Freshly fallen, intact leaves are only slowly colonized by oribatid mites, but numbers increase when leaves become fragmented and enriched with fungi and bacteria [7]. During the fragmentation process structural differences of leaf litter species diminish, and this may have contributed to the lack of effects of leaf litter species and diversity on the diversity of oribatid mites.

The generally weak response of oribatid mite community structure to leaf litter and root identity and diversity suggests that most oribatid mite species are weakly linked to traits of plant (tree) species, even though microorganisms associated with these plant species and serving as food for oribatid mites likely differed [18,71]. Litter mass and microbial biomass explained community patterns of oribatid mites to a certain degree, but direct effects of leaf litter and root chemistry and architecture apparently are of minor importance for structuring oribatid mite assemblages.

Stable isotope data suggest trophic niche differentiation among oribatid mite species, and food choice experiments have shown that different oribatid mite species prefer certain fungi, however, with many species preferring the same fungal species and with only few fungal species being entirely rejected [33,72,73]. The low degree of food resource specificity may enable oribatid mites to cope with a wide range of microhabitats and explain their low responsiveness to manipulations of litter species, but leaves the local coexistence of species elusive. The lack of trophic specialization further is supported by the lack of effects of root species on oribatid mite abundance and community structure in our study as the trees investigated are associated with different mycorrhizal symbionts (ectomycorrhizal vs. arbuscular mycorrhizal fungi).

Interestingly, and in contrast to our second hypothesis the proportion of parthenogenetic oribatid mite species did not differ between litter species. Surprisingly, root species affected the proportion of parthenogenetic individuals with highest proportions in the presence of beech roots. The proportion of parthenogenetic individuals and species has been proposed to increase with the amount of organic material present and to peak in forest soils with thick organic layers [38]. Although only marginally significant, this also was true in the present study as the proportion of parthenogenetic individuals tended to increase with the amount of remaining litter mass. The effect of root species on the proportion of parthenogenetic oribatid mite species was most pronounced in beech, thus a higher resource input via root exudation might have shifted the oribatid mite community towards parthenogenetic species in presence of beech roots which have been shown to strongly impact soil biota presumably due to high amounts of rhizodeposits [31,74]. This pattern fits predictions of the structured resource theory of sex which posits that parthenogenetic species flourish if resources are in ample supply [75].

The mechanisms structuring oribatid mite communities remain puzzling, but it appears that identity and diversity effects of plant species via leaf litter or roots only play a minor role. In our study, however, roots of the surrounding mature oak trees may have overridden species-specific root effects of the investigated seedlings by homogenizing the soil, thereby underestimating the importance of root-derived carbon resources. Although some studies indicate that plant identity and plant diversity in fact are structuring oribatid mite communities, these effects typically were weak and often restricted to only certain taxa or were only present at one of several sampling dates [44,45,47,52,70,76]. Presumably, small-scale differences in abiotic soil habitat properties are more important for structuring oribatid mite communities than litter identity and diversity. By manipulating the thickness of organic layers in a field experiment, Nielsen et al. [11] showed that small-scale habitat heterogeneity resulted in an increase in species richness of oribatid mites, and the same was true for collembolans and nematodes. In another study, Ducarme et al. [77] provided evidence that soil porosity and pH were main determinants for oribatid mite abundance, species richness and community structure suggesting that physical habitat characteristics might be more important drivers of oribatid mite communities than food resources. As plants affect soil physicochemical characteristics such as pH, moisture, porosity or humus form, plant cover might shape communities indirectly. Soil formation processes, however, are slow taking decades or centuries and thus, consequences are difficult to detect in experimental studies even if they last for several years as in the present study.

## Supporting information

S1 Appendix Names of oribatid mite species, their abbreviations as used in Fig 4, their reproductive mode and their relative abundance (% of total). (XLSX)
